# Status of diagnosis and therapy of abdominal aortic aneurysms

**DOI:** 10.3389/fcvm.2023.1199804

**Published:** 2023-07-28

**Authors:** Jinping Lin, Shuwei Chen, Yuanyuan Yao, Min Yan

**Affiliations:** ^1^ Second Affiliated Hospital, School of Medicine, Zhejiang University, Hangzhou, China; ^2^ Department of anesthesiology, The First People's Hospital of Fuyang, Hangzhou, China

**Keywords:** abdominal aortic aneurysm, pathogenesis, diagnosis, endovascular aneurysm repair, complication

## Abstract

Abdominal aortic aneurysms (AAAs) are characterized by localized dilation of the abdominal aorta. They are associated with several serious consequences, including compression of adjacent abdominal organs, pain, treatment-related financial expenditure. The main complication of AAA is aortic rupture, which is responsible for about 200,000 deaths per year worldwide. An increasing number of researchers are dedicating their efforts to study AAA, resulting in significant progress in this field. Despite the commendable progress made thus far, there remains a lack of established methods to effectively decelerate the dilation of aneurysms. Therefore, further studies are imperative to expand our understanding and enhance our knowledge concerning AAAs. Although numerous factors are known to be associated with the occurrence and progression of AAA, the exact pathway of development remains unclear. While asymptomatic at most times, AAA features a highly unpredictable disease course, which could culminate in the highly deadly rupture of the aneurysmal aorta. Current guidelines recommend watchful waiting and lifestyle adjustment for smaller, slow-growing aneurysms, while elective/prophylactic surgical repairs including open repair and endovascular aneurysm repair are recommended for larger aneurysms that have grown beyond certain thresholds (55 mm for males and 50 mm for females). The latter is a minimally invasive procedure and is widely believed to be suited for patients with a poor general condition. However, several concerns have recently been raised regarding the postoperative complications and possible loss of associated survival benefits on it. In this review, we aimed to highlight the current status of diagnosis and treatment of AAA by an in-depth analysis of the findings from literatures.

## 1. Introduction

Abdominal aortic aneurysms (AAAs) are characterized by a diameter which measures 1.5-fold that of the normal descending aorta (between the diaphragm and common iliac artery bifurcation); anatomically, this involves all three classical layers (1–3). Although these are usually single spherical or prismatic aneurysm, multiple aortic aneurysms of abdominal aorta can be detected occasionally (3). AAA is considered as an age-associated condition, having a prevalence of 1.2%–4% in the population aged over 50 years (4) and up to 8% among males aged over 65 years (mostly those suffering from hypertension and heart disease) (5–8); however, this disease can also affect the young (9). The male-to-female gender ratio is approximately 10:3 (5, 10). Data from studies suggest that without any surgical intervention, the 5-year risk of AAA rupture increases with an increase in aneurysmal diameter; the risk increases by less than 1%, less than 5.3%, and more than 6.3% with diameters of 5 cm, 5.5 cm, and 7 cm, respectively (11–13). Based on the relationship between aneurysm rupture possibility and diameter (14), investigators consider AAAs with diameters of ≥5.5 cm to be more lethal (15, 16). The most critical consequence is aneurysm rupture, which leads to hemorrhagic shock; this causes approximately 2.8 to 100,000 deaths (mortality rate: 50%–80%) globally each year, with an increasing tendency in recent years (9, 14, 17). However, failure to detect the condition in the early stages occasionally causes it to be overlooked (12, 18). Most often, physicians diagnose the disease while examining patients for other reasons (19–21). Owing to the significant health risks, it is essential to urgently determine its etiology and pathogenesis. Precautions, timely diagnosis, and appropriate treatments are needed to improve patients' health and increase their chances of survival.

## 2. Etiology and pathogenesis

The aortic intima has a thick underlayer of endothelium, beyond which are multiple layers of elastic membrane connected with media. Elastin and collagen are the principal structural materials of the aortic wall, and constitute the media in conjunction with vascular smooth muscle cells (SMCs). Adventitia is thin, consists of connective tissues and translates to surrounding loose connective tissue gradually (13, 22–24). Elastin, the primary component that withstands arterial pressure, forms a folded cribrosa that stretches 70% beyond its natural length in response to external force, which leads to longitudinal/cross-sectional retraction and maintains the normal aortic diameter and shape (25). Collagen functions to maintain the tensile strength of the aortic wall, where types I and III collagen consist of three spirally bound polypeptide chains which offer considerable elasticity (which is twenty-fold greater than that of elastic fibers), but minimal extensibility (3). Under normal circumstances, both elastin and collagen work in unison to maintain the flexibility and pliability of the aorta. In the event of increased pressure, collagen provides support spirally to enhance the resistance of elastin against pressure, which synergistically reduces exceptional dilatation of the abdominal aorta, thereby preventing the development of AAA. In this context, multiple factors are involved in the occurrence and progression of AAA (26, 27) whose pathogenesis therefore remains considerably complicated (28, 29).

Previous studies suggested that AAAs may be caused by the evolution of arteriosclerosisas they are mainly prevalent in the elderly and most commonly occur at a site below the origin of the renal arteries (which coincides with the site for atherosclerosis) (13, 30). However, there is a new understanding that AAAs and atherosclerosis are might not directly related with each other (31), which isn't accordant with the conclusion in literatures published previously. Furthermore, the relationship between the degree of arteriosclerosis and diameter of the aneurysm remains unclear (32). Notably, investigators have found the incidence of AAAs to be significantly higher among patients with carotid and lower limb atherosclerosis (33). In addition, approximately 44% of patients who undergo open repair (OR) have associated arteriosclerotic diseases including carotid atherosclerotic plaques and coronary heart disease (5, 6, 10). In this context, collagen is degraded by collagenase, which is activated by naked SMCs after detachment of atherosclerotic plaques (23). It is worth mentioning that the aortic wall between the level of origin of the renal arteries and aortic bifurcation (where the reflex pressure is greater) is exposed to hardening factors for a prolonged period of time owing to narrowing of the iliac arteries (by atherosclerotic plaques and mural thrombi) (13). Disparities in nutrient supply may therefore lead to different outcomes in different vessels (such as the carotid artery and those of the lower extremities). As the abdominal aortic wall mainly obtains nutrients from the blood by diffusion, atherosclerosis inevitably impairs nutrient diffusion and leads to necrosis of the arterioles and media and impairs repair capability (34).

Histopathologically, AAAs are characterized by an inflammatory response with infiltration by a large number of macrophages and proinflammatory cytokines; however, the precipitating factors remain unclear (13, 35, 36). An abundance of immunoglobulins is also observed, implying an autoimmune response (13, 35, 37, 38). Interleukin-1β and tumor necrosis factor-α that are secreted by macrophages play an essential role in the inflammatory process; they stimulate the production of matrix metalloproteinases (MMPs) and weaken or destroy the middle aortic layer (39–41). However, the inflammatory signaling pathway involved in the occurrence and development of AAA needs to be thoroughly investigated (26).

In this context, studies indicate the role of certain structural changes and defects in the pathogenesis of AAA. In view of the conical anatomical structure of the aorta, the pressure exerted on the aortic wall increases in the craniocaudal direction with a progressive reduction in elasticity from the proximal to distal end (42). The degree of amplification of blood pressure from the aortaventralis to the peripheral arteries (bilateral common iliac and renal) is determined by the ratio of the diameters of the aorta and peripheral arteries. The reflected pressure is considered optimal when the total diameter of both common iliac arteries equals 1.1–1.2-fold that of the aorta. This ratio gradually declines with age, reducing to 0.75-fold around the age of 50 years (3, 43). Notably, an aneurysm is likely to occur when the elastin disintegrates to a below a thickness of forty layers (44). In the human body, the half-life of elastin and the peak age for aneurysm formation coincide at the age of 70 years; this phenomenon has been found to be consistent. Quantitative analysis has shown that elastic fibers account for 35% of the dry weight of the normal aortic media, while they comprise only 8% in aneurysms (45). In this context, SMCs produce and synthesize collagen and elastin when stimulated by the concussive force induced by impulse pressure (46, 47). Stiffness of the abdominal aorta occasionally weakens the stimulation; this stiffness is mainly caused by a reduction in the production of elastin and collagen or inappropriate apoptosis of SMCs (22, 48, 49), which are replaced by fibrous connective tissue (13, 23). However, the factors initiating such changes remain unclear.

The genetic factors associated with the development and progression of AAAs are complex and largely unknown (13, 50, 51). Studies have shown that 15%–19% of patients are close relatives of those with a known history of aneurysms. However, on comparing the family history of hundreds of patients in a study cohort with that of the control group, the proportion was found to be only 1%–3% (52, 53). In this context, the risk of AAA occurrence has been found to be high among siblings (54), especially in monozygotic twins (55). The inheritance of AAA is mainly associated with autosomal genes relevant to collagen metabolism and demonstrates autosomal dominant heredity (56, 57). It is also reported that patients with Marfan syndrome are more likely to develop thoracoabdominal aortic aneurysms and aortic dissection (58). Genetic variants participating in AAA progression may be related to abnormalities in the quantity and activity of collagenase, elastase, MMPs, and other enzymes that directly or indirectly weaken the aortic wall by increasingly inactivating and degrading matrix structural proteins (49). However, corresponding available data are limited and conflicting (35). In view of the sporadic nature of base mutations, studies in humans that included large sample sizes have not been able to clearly define the role of genetics. A recent study that included cases with genetic mutations suggested that the type III pro-collagen gene plays an essential role in AAA pathogenesis; although such mutations only found in a few patients, the replacement of a single amino acid residue can cause profound qualitative changes. In terms of content and elastase (neutrophil and smooth muscle elastase) activity, the impact is greater than that of aortic occlusion. After the onset of arteriosclerosis, SMCs in the arterial wall are stimulated to produce elastase in large quantities, laying the foundation for aneurysm formation. Mutations in the haptoglobin gene on the long arm of autosome 16, and its adjacent cholesteryl ester (transfer protein) gene, are also known to play a role in AAA pathogenesis. The expression of the haptoglobin α1 allele is considerably up regulated in these cases, leading to plentiful synthesis of haptoglobin and degradation of elastin; this affects the integrity of elastic connective tissues in the aortic wall. In this context, abnormal expression of the cholesteryl ester (transfer protein) gene influences lipid metabolism, lowering concentrations of high-density lipoprotein (considered as a protective factor) and increasing levels of triglycerides and low-density lipoproteins; this increases the hazards of cardiovascular disease (59). Notably, absence of the α1-antitrypsinogen gene is also a risk factor, as this molecule is the most predominant inhibitor of elastase. In this context, findings suggest that balloon remodeling of the aortaventralis may activate collagenase. Increasing evidence from literature suggests that the metabolism of MMPs may play an essential role in the pathogenesis of AAA (40, 60). In 1984, Tilson et al. (61) found that deficiencies in copper metabolism in a mice AAA model reduced the activity of lytic oxidase (a copper-containing MMP) that acts as a bond, connecting collagen and elastin. Reduced aortic elastic tissue and abnormal copper metabolism are also observed in patients with Menkes syndrome. Studies have also found the activities of zinc-related MMP-3 and MMP-9 to be increased in AAA; notably, these enzymes are primarily responsible for the degradation of matrix components in the aortic wall (41, 62).

In addition to the aforementioned factors associated with the development of AAA, numerous other risk factors including smoking (20, 63), iatrogenic factors, and hypertension (64) also influence AAA progression. Studies performed over the past two decades have demonstrated that the morbidity of AAA gradually increases with cigarette consumption (6). Various toxic ingredients and gaseous substances may convert methionine into methionine sulfoxide, leading to decreased expression of α-smooth muscle actin, increased activity of proteolytic enzymes, accelerated degradation of elastin, and reduced strength of the aortic wall (39, 63, 65). In this context, data suggest that average smokers are 4–8 times more likely to suffer from aneurysms than non-smokers (6, 20, 66) and chain smokers are 14 times more likely to die from the condition as compared to average smokers (67–69). There is growing speculation that patients who undergo an exploratory laparotomy are more likely to experience aneurysm rupture within the first 36 h of surgery, which is based on the fact that surgical procedures disrupt the dynamic balance between catabolism and anabolism of matrix proteins, causing a considerable increase in elastase activity in the aorta. Ironically, no matter how plausible this view may seem, it is dubious due to lack of clinic data or basic research result support. However, it remains debated as to whether it is beneficial to treat AAA before or simultaneously with laparotomy. In this context, recent studies on animal models of AAA evaluated the impact of hypertension (especially systolic hypertension, which is a basic condition for aneurysm development) on the formation of aneurysms; no definite conclusion could be drawn as to whether hypertension plays a role in the formation of aneurysms or merely contributes to expansion of the weakened aortic wall in humans (64, 70, 71). It is certain that AAAs is weak associated with systolic blood pressure while stronger associated with diastolic blood pressure and mean arterial pressure, and arterial rigidity that protects against AAAs might account for this phenomenon (72). As the rate of dilatation in the transverse axis exceeds that of the anteroposterior axis, the cross-section of an AAA is likely to be oval; this is consistent with the fact that AAAs often rupture laterally. However, little or no information is available in the literature regarding the rate of expansion in vertical diameters.

In summary, the risk factors associated with AAAs include one condition of insufficiency and eight conditions of excess; these include hypertension, hyperlipidemia, hyperglycemia, hyperuricemia, high body mass index, increased blood viscosity, older age, psychentonia, and physical inactivity. Other rare causes include congenital aortic dysplasia, syphilis, acute or chronic infection, aortitis, cancer, and alcoholism, among others (1, 73–75).

## 3. Classification

Two frequently used approaches are used applied for the classification of AAA. One is based on the structure of the aneurysms. The aneurysm is considered to be a true aneurysm, if all three layers of its wall are structurally intact. Conversely, a false aneurysm has an incomplete wall structure that partially consists of arterial intima and fibrous tissue. The blood flow in the aneurysm lumen communicates with the true lumen of the aorta via the entry tear. Traumatic, infectious, and anastomotic pseudoaneurysms are all false aneurysms; similar to AD, blood flows in the space between the aortic intima and media creating aortic wall distention. Thoracoabdominal aortic aneurysms and suprarenal AAAs represent other clinically entities; these are classified based on the location of the aneurysm which occupies the space above the origins of the renal arteries (76). The clinically common infra-renal AAA is located below the origins of the renal arteries and above the bifurcation of the abdominal aorta (1, 77). In the latter category, different lengths of normal aortic wall are observed at the proximal and distal ends; this proves to be favorable for surgical procedures (78). Notably, irrespective of category, AAAs demonstrate both elastic layer fracture and disappearance of the intima (25, 79); chronic or acute thrombogenesis (80) and/or ulceration is predominantly found in the aneurysm and/or along the aortic wall.

## 4. Clinical features

AAAs patients with symptoms often present with diffuse nonspecific abdominal and/or lower back pain. Also, patients may claim that they feel pulsations in their abdomen and see a pulsatile mass in their abdomen. The sensitivity of detection of AAAs by abdominal palpation increases with the diameter of the lesion, and depends inversely on the size of the abdominal waistline (81). In this context, the abdominal pulsation may be palpated in individuals with a lower body mass index or lesser belly fat and even in women with a normal or high body mass index (usually in those with diastasis recti abdominis consequent to parturition). Auscultation offers a suitable method in approximately 50% of cases with a systolic murmur (82); in addition, a few patients have weak or no pulses in the femoral or dorsalis pedis arteries (83, 84). However, most cases have no signs or symptoms in the early stage, even before aneurysm rupture (85).

The symptoms associated with AAAs are non-specific and involve compression, pain, and arterial embolism, among others. Abdominal pain, ileus, bowel necrosis, icterus, and left-sided renal insufficiency may be observed in cases with compression of the duodenum, bile duct, and urinary system (16, 86). Pain, listed as the fifth most vital sign by the World Health Organization, mainly occurs around the umbilicus and middle or upper abdomen; subtle differences are observed depending on the location of the aneurysm (16). Back pain is induced by impingement on the lumbar spine (87). Additionally, thrombosis or ulcerative debris may cause lower extremity embolism, which leads to pulselessness, acroparalysis, paresthesia, necrosis, and possibly infection (83, 84). The mesenteric artery can also be blocked, leading to intestinal ischemia, signs of peritoneal irritation, hypotension, septic shock, renal artery obstruction (caused by infarction of the corresponding part of the kidney), severe pain on percussion, and hematuria (88). Tears form when the pressure in the aortic lumen exceeds that tolerated by the expanded aorta; this results in typical symptoms such as sudden severe abdominal pain (18), hypotension, and shock (86, 89). Notably, newly growing severe abdominal or back pain, and severe hypertension often indicate that the aneurysm is on the verge of rupture; these are occasionally the initial complaints on presentation in some cases (12, 18). In this context, the clinical features vary slightly between the five different types of aneurysm rupture.

In the most critical cases, blood rushes into the abdominal cavity when the point of leakage reaches the aortic anterior wall, which causes severe hemorrhagic shock, leading to death within a short time even before first-aid can be delivered (90). Therefore, the actual incidence and morbidity are higher than the values recorded in the clinic (9). The flow of blood into the retroperitoneal cavity is commonly characterized by a relatively slow velocity, which occurs when the tear reaches the posterolateral aortic wall. The associated symptoms and complications can extend for several days, leaving a margin of time for medical assistance. However, distinguishing AAA from conditions such as acute pancreatitis, mesenteric vascular embolism, peptic ulcer perforation, and aortic dissection can pose challenges due to overlapping clinical presentations (17). The bleeding is occasionally localized, leading to coagulation and subsequent formation of a block mass. Such patients may experience abdominal pain, fever, mild to moderate anemia, and unpredictable repeat ruptures and can be misdiagnosed with inguinal hernia and femoral neuropathy, among others. The inferior vena cava may also be involved in 1% cases; this leads to vena caval obstruction, which may contribute to peripheral edema and the development of aorto-venous fistulas that are characterized by continuous murmurs, high cardiac output, and heart failure, among other symptoms (1). Rupture into the intestinal lumen leads to the development of abdominal aorto-enteric fistulas, which manifest as intermittent gastrointestinal bleeding and remittent fever which lasts for several days or weeks (91). This diagnosis is supported or confirmed by the results of blood cultures, which show the organisms to be consistent with normal intestinal flora. Intestinal bacteria rarely disseminate via the blood to cause suppurative arthritis and localized infection of the lower extremities (92).

## 5. Diagnostic and imaging features

As the clinical signs and symptoms associated with AAA are non-specific, conditions with similar symptoms (such as renal colic) account for more than 20% of the total misdiagnosed cases; celiac disease and myocardial infarction are also diagnosed (16). The presence of severe abdominal pain, pain on percussion, and microscopic hematuria may mislead clinicians, leading to a diagnosis of urinary calculi and renal colic, which may be due to the lack of awareness of the symptoms (which are usually observed when the upper urinary system is encompassed by blood or the renal artery opening has ruptured). Abdominal discomfort often leads to misdiagnosis of the case as being one of celiac disease, gastrointestinal bleeding and perforation, intestinal obstruction, cholelithiasis, acute suppurative cholangitis, pancreatitis, and sigmoid diverticulitis, among others. The condition is also likely to be overlooked at the general surgery department when a large retroperitoneal hematoma that develops consequent to AAA rupture increases pressure on the vulnerable inguinal area and contributes to incarceration of hernial contents. Another condition that needs to be appropriately diagnosed is acute myocardial infarction, because patients with AAAs often have atherosclerosis of the coronary artery (93). However, many patients with myocardial infarction have a history of recurrent angina pectoris, and the location of the pain site differs slightly. In myocardial infarction, the pain mostly occurs behind the sternum and spreads to the neck and left arm which can be alleviated by nitrates and morphine. In contrast, the pain associated with AAA rupture involves an extensive area, and analgesics such as opioids are ineffective. Continuous electrocardiographic monitoring for observing specific changes, serum myocardial enzyme profiling, and imaging tests may provide evidence for an appropriate diagnosis. Blunt abdominal trauma can also be differentiated by considering the patients' medical history.

In summary, the diagnosis of AAA relies heavily on the combination of medical history-taking and clinical features, especially in areas where the availability of imaging facilities is limited. There is an urgent need to improve clinical facilities and increase awareness on the condition to reduce the rate of initial misdiagnosis or missed diagnoses, and to shorten the mean time from symptom onset to a definite diagnosis. As there are no specific blood markers for AAA, imaging examinations can be performed to obtain a definite diagnosis once it is suspected via physical palpation (94). Therefore, various imaging techniques have been developed and utilized for the diagnosis of early-stage AAAs, which also have their respective advantages and limitations (94).

### 5.1. Abdominal radiographs

Radiographs are used for imaging worldwide which require moderate expenditure and are time-saving. The diagnosis of AAA can be confirmed in cases where the aneurysm is calcified and a typical egg-shell-shaped calcification shadow appears on the radiograph. However, at least a quarter of such patients do not have these typical indicators. Although a calcified shadow of the aneurysm wall is observed in a few cases, absence of a calcified shadow cannot negate the presence of an AAA. The aneurysms' size and shape may also be approximately determined in two-thirds of patients (95).

### 5.2. Ultrasonography

Ultrasound is currently an effective and valuable diagnostic tool for the diagnosis of AAAs, with a sensitivity of 95% and specificity nearing 100% (96); it is suitable for population screening, as it can provide a clear picture of the vascular wall and mural thrombus (96, 97). It also offers the advantages of being user-friendly, time-saving, acquirable using a simple apparatus, involving no radiation, and low-cost. Ultrasound analysis has offered valuable insights into increase of aneurysm diameter. The previous researches suggest that the diameter increases by 3.8 mm/year on an average. It is documented that the dilatation of small AAAs cannot be predicted appropriately by imaging tools other than ultrasound (16). However, it is important to highlight that in some cases, inexperienced sonographers may exert excessive pressure on the abdomen, causing more serious situation when scanning ruptured AAA without symptoms.

### 5.3. Computerized tomography angiography

Computerized tomography angiography (CTA) is the most common imaging technique used to accurately identify AAAs; it is also superior to ultrasound in determining the positional relationship between AAAs and the adjacent structures and for postoperative evaluation (96, 98, 99). In addition, CTA may demonstrate the lateral thrombus clearly, making it superior to traditional digital subtraction angiography in terms of measurement of the diameters of the aneurysms and iliac arteries to obtain key information for surgical planning (98). It also is valuable in the detection of postoperative complications (96). Reconstruction of a three-dimensional image of the artery allows for a comprehensive visualization of the arterial structure, aiding in the assessment and understanding of AAA morphology and its surrounding anatomy. The disadvantages of CTA include the involvement of higher costs, the need for a longer operating duration than certain other imaging methods, the involvement of radioactivity, and the need for administration of an intravenous contrast medium (which may worsen renal dysfunction and lead to a brief sensation of heat or a metallic taste in the mouth) (100).

### 5.4. Magnetic resonance imaging

The performance of magnetic resonance imaging is comparable to that of ultrasonography and CTA (101, 102). However, it is associated with the disadvantage of being expensive and time-consuming. Nevertheless, technological advancements are expected to reducing the image processing time. Patients with AAAs usually have other accompanying vascular diseases that limit the use of magnetic resonance imaging. For instance, some of these patients may have undergone percutaneous transluminal coronary intervention or stent implantation in the femoral, subclavian, or carotid artery.

### 5.5. Magnetic resonance angiography

Magnetic resonance angiography offers accurate detection of AAAs. However, it is not widely applied, as the actual width of the aneurysm may be obscured by a mural thrombus and the procedure is costlier which requires longer imaging times. The equipment requirements are also higher. However, it offers the advantage of not requiring the use of contrast agents, making it especially suitable in cases where these are contraindicated (including patients with allergic reactions to intravenous contrast and renal failure). In addition, patients are not exposed to ionizing radiations during imaging (103).

### 5.6. Angiography

Angiography is the only real-time display imaging modality that can accurately identify the exact location and degree of vascular lesions; however, the actual size of the AAA cannot be determined because of the lateral thrombus (104). Unlike traditional angiography, digital subtraction angiography eliminates bone and soft tissue shadows by processing the digitized image information via a computer to allow clear visualization of the vessels. However, it is not the preferred screening method due to its invasive and potential to cause anaphylaxis or delayed hypersensitivity reactions and requires the patient to be stationary for a long duration. It is reserved for cases where further detailed characterization of an aneurysm is required for intervention, as in the case of preoperative embolization of an accessory renal artery prior to endovascular aneurysm repair. Notably, postoperative immobilization is not suitable in cases with disorders of consciousness.

## 6. Management

The management of AAAs currently poses a considerable challenge, as aortic aneurysms cannot be healed naturally. Vascular surgery methods and material technology have improved continuously over the past 30 years, allowing an increasing number of patients (especially those with a poor general condition) to obtain treatment (either OR or endovascular therapy). The preoperative mortality rate has decreased from 15% in the 1950s to approximately 5% (and even 2%–3% based on current reports) (105). The survival rate (as recorded over the past 5 years) exceeds 60% and is as high as 79% in patients aged less than 70 years (106). The best approaches for preventing AAAs include improvement of body composition by adoption of a healthy diet (low fat and low cholesterol diet) (10, 105), performance of regular exercise (10, 19), control of the heart rate by beta-adrenergic blockade (107–109), and avoiding smoking and other unhealthy practices (67). It reported that individuals with large aneurysms might be at greater risk of an adverse event during exercise, while training at low and moderate intensities may be protective for aneurysm expansion without the information about the size of aneurysms (110). Therapeutic measures include medical therapy, OR, and endovascular therapy.

### 6.1. Medical therapy

Effective control of hypertension, cardiac rates, blood glucose levels, serum lipid levels, atherosclerosis, and other parameters may delay the rate of expansion. For instance, taking statins, dipyridamole, and metformin to prevent secondary thrombosis and improve lower limb ischemia can also prevent the occurrence and rupture of AAA to a certain extent (48, 70, 111). Although there is still no certain cure for AAA (112, 113), some promising therapeutic agents have been reported. Apoptosis inhibitors can delay progression by suppressing the inflammatory response in the aneurysm wall (48). In addition, MMP inhibitors alleviate expansion by slowing down extracellular matrix remodeling, in which statins may ease the inflammatory response in the aortic wall (114), and metformin may limit AAA expansion and rupture (115). Unfortunately, although certain agents may inhibit the progression of AAA, they are ineffective in reversing the condition. Only several beneficial agents have been reported to be effective in slowing down artery dilation in animal studies (47, 116, 117). As AAA rupture is an acute condition, early detection is extremely important. In cases demonstrating a tendency to rupture, emergency procedures need to be performed immediately (1). Patients who are intolerant to surgical treatment should actively receive appropriate conservative management.

### 6.2. Surgical treatments

Surgery remains the first-line treatment in AAA. Based on recent reports, the 5- year survival following routine elective surgery was more than 70% (106) in patients with heart disease, cerebrovascular accidents, malignant tumors, and other conditions that are leading causes of death (118, 119). The surgical methods include traditional OR and endovascular aneurysm repair (EVAR).

AAA was first resected and replaced successfully by an allogeneic aorta in 1951. In addition, a case of traumatic aneurysm was treated similarly in China in 1956. The rapid development of materials science has allowed the gradual replacement of OR by aneurysmectomy and artificial vascular graft placement. The postoperative mortality from OR ranges between 1.2% and 8.2% (118, 120–122). The surgical principle is based on the positional relationship between the aneurysm sac and renal artery and the blood supply to vital organs. In cases where the AAA is located below the origin of the renal artery, a transperitoneal or retroperitoneal approach is needed to expose the abdominal aorta below the renal and bilateral iliac arteries. In order to dissociate and block the arteries after heparinization, the aneurysm sac is incised, arteries are ligated instantly, thrombi and atheromatous debris are removed, and a tube graft or a bifurcated graft is implanted. After completing the anastomosis, the graft is wrapped by the incised aneurysm sac and sutured. The blood supply of the left colon may be ligated or the inferior mesenteric artery may be sutured to the side wall of the graft. If the distal anastomosis is located distally below the common iliac artery bifurcation, the blood supply of at least one internal iliac artery needs to be preserved. A thoracoabdominal incision is optimal for suprarenal AAA. In cases requiring implantation of an artificial graft, the procedure endeavors to shorten the duration of visceral ischemia and avoid organ function damage caused by ischemia; this is achieved by rapidly connecting the celiac, superior mesenteric, and renal arteries with the graft. Recent years, with the rapid development of minimally invasive technology and stenting techniques, EVAR including complex EVAR are widely recognized and applied. However, the role of traditional OR is still irreplaceable, especially in cases with complex anatomics or coexisting disease processes that prohibit them from an endovascular repair.

### 6.3. EVAR

EVAR offers an alternative to traditional interventions for AAAs (16) and was first performed in the 1990s (123, 124). It involves the placement of a stent in the dilated aorta to exclude the aneurysm from the aortic circulation and reduce the risk of rupture (125). In general, EVAR involves the transportation of a metal scaffold into the aneurysm lumen via the femoral artery using a special delivery device, which is then connected to the aortic wall using the elastic features and hooking attachments. In patients with AAA involving the iliac artery, blood flow needs to be maintained in at least one of the internal iliac arteries to ensure adequate blood supply to the pelvic organs and gluteal muscles. Alternatively, a bypass may be created between the internal and the external iliac artery while performing EVAR.

The indications for EVAR are similar to those for traditional OR, which include an aneurysm diameter of >5.5 cm, complaints of discomfort, and an increase in aneurysm diameter (by 5 mm biannually or at least 10 mm annually) (125). There are certain other stringent criteria related to the aneurysm, such as that the diameter of the aneurysm sac should measure <7 cm, external iliac artery diameter should range between 7 and 14 mm, and iliac artery angulation should exceed 90° (if not 90°, it is essential that diffuse calcification is absent) (126). During stratification of the risks of stentgraft failure, the points-scoring system is used for emphasizing the value of the aortic proximal part, which involves the length, diameter, angulation, and extent of calcification of the proximal normal aorta, and the presence of thrombi is also considered. Ideally, the length, diameter, and angulation should be >15 mm, <30 mm, and >120°, respectively, and the calcification should not extend beyond half of the perimeter and no thrombi should be present. EVAR is contraindicated in cases where the aneurysm involves both iliac arteries or the hypogastric artery with one occluded contralateral artery. However, the mentioned criteria are absent in acute inflammatory AAA.

EVAR is widely believed to be more suitable for patients with poor general condition for those aged over 80 years; those suffering from obesity, diabetes, heart failure, and respiratory or renal insufficiency; and those having class III or IV physical status as per the American Society of Anesthesiologists classification (127). Patients with complicated AAAs therefore initially require invasive surgery for administration of minimally invasive treatment. In this context, the chimney and window techniques have been developed and implemented over time. The hybridized technique of OR and EVAR has also been performed successfully in patients with a poor general condition and visceral artery involvement who are not suitable for OR.

There are tough situations including juxtarenal abdominal aortic aneurysms (JRAAA) and thoracoabdominal aortic aneurysms (TAAA), where traditional EVAR is failed to meet the demands for treatment due to it requires sufficient perfusion of splanchnic artery branches when isolates aneurysm cavity from the aorta. Therefore, Ch-EVAR, F-EVAR, B-EVAR and so on applied making complicated AAA successfully treated by interventional therapy. Ch-EVAR, refers to extract subordinate stent rom an essential branch artery parallelly to the main covered-stent, is an effective alternative method to traditional EVAR, especially for JRAAA patients who are not suitable for open surgery due to high surgical risk (128). When perform F-EVAR, a specifically designed stent according to the diameter and location of branch artery is required (129, 130). Differing from Ch-EVAR and F-EVAR, branch stent-graft has real branches which can stand greater longitudinal pressure and is the ideal device for AAA with poor aortic neck anatomy (131). Currently, an increasing number of high-level evidence comparing complex EVAR and OR is accumulating and being updated.

However, there are some concerns regarding the possible loss of survival benefits with EVAR (132). Although it provides similar early survival advantages as OR, with similar or lower operative mortality (118), a lower injury rate, shorter hospital stay, and rapid recovery (125, 133–136), it offers similar or poorer long-term survival due to the incidence of postoperative complications (which is as high as 16%–30%) (137–139). Therefore, long-term follow-up and re-intervention are needed to identify systemic complications and manage graft-related complications (99, 118). Other than conversion to laparotomy, approximately one-tenth of cases experience systemic complications, which are mainly related to ischemia (induced by arterial thrombosis, embolism, detachment, or obstruction resulting from stent displacement). Lower limb ischemia is the most common type of ischemia complications after EVAR, and may manifest as aches, paresthesia, intermittent claudication, and a weak femoral pulse. Other complications include, but are not limited to, intestinal ischemia and paraplegia.

Complications associated with implanted devices are variable and considerably common (Table 1) (136). The most frequent complication is endoleak (140). Based on the origin, endoleaks may be categorized into five types, namely, attachment site leak, retrograde blood flow from aortic branches, device failure, graft porosity, and endotension with variable CTA features. In cases where imaging demonstrates discontinuity of suture points and/or metallic frames, the incidence of major suture breaks and metal ring fractures is 5.5% (141). Device migration refers to the distance by which the device moves (by more than 10 mm at the centerline or more than 15 mm at either the anterior or posterior aortic margin) and is detected in 1%–10% of cases (142). Insufficient overlap between the device and aneurysm and changes in the aneurysm size and hemodynamic force may contribute to its development (90). In addition, data from studies show that 0.5%–11% of patients experience graft thrombosis and occlusion (129) owing to migration, kinking, and inadequate size of the device; irregular intake of medication or incorrect dosages of anticoagulant drugs. Additionally, 2%–4% of patients may experience device kinking or angulation when the residual aneurysm sac gradually reduces in size, which makes the diameter of the distal aortic segment exceptionally small and the angle of the proximal aortic part exceptionally large (133). This complication is frequently localized to the stent-graft limb and can be managed by implanting an additional graft or stent in the primary device following percutaneous transluminal angioplasty. Stent-Graft infection, a rare complication with an incidence of less than 1% and high mortality (69), is often caused by surgical procedures and infections of other distant organs, which presents with fever, high white blood cell counts, and backache. In serious cases, EVAR performed via the arteria cruralis may lead to pseudoaneurysm formation (tear of the arterial wall with collection of blood, that is contained by the adventitia or surrounding perivascular soft tissues), thrombosis, dissection, hematoma, infection, and lymphocele, among others. Careful arterial puncture under ultrasound guidance may help reduce the incidence of such complications to a considerable extent. Additionally, renal artery occlusion (143), pelvic ischemia (144) are rare not absent clinically.

**Table 1:** Complications and relevant CTA findings following EVAR (69, 118, 133, 135–139, 142).

Complication	Type of complication	CTA features	Incidence
Systemic	Ischemia	Limb ischemia; Bowel ischemia; Spinal cord ischemia.	9%
	Access site complication	Pseudoaneuysm; Thrombosis; Dissection; Hematoma; Infection.	3–5%
Endograft device-related	Endoleaks (five types)	Contrast extravasation in aneurysm sac	15–30%
* *	*Attachment site leak*	*Centrally located, adjacent to one attachment site of the endograft*	
	*Retrograde blood flow from aortic branches*	*Peripherally located, near the origin of the involved vessels*	
	*Device failure*	*Centrally located, close to the attachment site of the endograft*	
	*Graft porosity*	*Hazy opacification around the stent-graft, without detectable source of endoleak*	
	*Endotension*	*Expansion of the aneurysm sac without signs of contrast extravasation*	
	Device migration	Movement >10 mm at the centerline or >15 mm at either the anterior or posterior aortic margin	1–10%
	Device kinking or angulation	More frequently localized at stent-graft limb	2–4%
	Graft thrombosis and occlusion	Non-enhanced concentric or eccentric thrombus along the internal wall of the endograft	0.5–11%
	Infection	Perigraft fluid collection, abnormal enhancement, air bubbles, and erosion into adjacent structures	<1%

## 7. Discussion and conclusion

AAA is more common than previously reported and presents considerable diagnostic and treatment challenges due to the absence of signs or non-specific symptoms in early stage. Studies and analysis on early detection including figuring out high risk group by simple and easy method such as blood-specific indicators should be applied on urgently. There are currently no treatments to reverse the occurrence and progression of AAA in humans. More efforts should therefore be made to study the mechanism of its development and identify methods for its prevention and non-operative treatment, coupled with building good living habits. Adequate awareness of AAA by patients is the prerequisite for seeing a doctor and performing treatments timely, so that relevant medical workers preaching relevant knowledge on AAA is beneficial. Similarly, it is essential to raise awareness of non-vascular clinicians and vascular surgeons' diagnostic and treatment-related skills. In addition, surgeons need to be aware of the advantages and disadvantages of OR and EVAR and endeavor to reduce complications (145). Improvement and innovation of minimally invasive methods for mitigating the adverse effects resulting from AAA is the eternal theme in the medical activity.
